# Innate immune alterations are elicited in microglial cells before plaque deposition in the Alzheimer’s disease mouse model 5xFAD

**DOI:** 10.1038/s41598-018-19699-y

**Published:** 2018-01-24

**Authors:** Antonio Boza-Serrano, Yiyi Yang, Agnes Paulus, Tomas Deierborg

**Affiliations:** Experimental Neuroinflammation Laboratory, Department of Experimental Medical Science, Lund University, Sölvegatan 19, BMC B11, 221 84 Lund, Sweden

## Abstract

Alzheimer’s disease (AD) is the most common form of dementia characterized by the formation of amyloid plaques (Aβ). Over the last decade, the important role of the innate immune system for the disease development has been established. Chronic activation of microglial cells creates a proinflammatory environment, which is believed to be central for the development of the disease as well as its progression. We used the AD mouse model 5xFAD to investigate if inflammatory alterations are present in microglial cells before plaque deposition. We applied mass spectrometry and bioinformation analysis to elucidate early microglial alterations. Interestingly, we found the cytokines IL1β and IL10 to be elevated in the 5xFAD brain after the formation of Aβ plaque at 10 weeks only. Using mass spectrometry analysis of microglial cells with bioinformation analysis, we found JAK/STAT, p38 MAPK and Interleukin pathways affected in microglial cells before plaque deposition at 6 weeks. At 10 weeks, GO analysis showed affected pathways related to interferon-gamma regulation and MAPK pathways. Our study points toward early inflammatory changes in microglial cells even before the accumulation of Aβ.

## Introduction

Alzheimer’s disease (AD) is a neurodegenerative disease affecting more than 24 million people worldwide and future projections predicts almost 50 millions people affected by 2030^1^. The two main hallmarks of the disease are the amyloid plaques (Aβ) and the intra-neuronal phospho-Tau aggregation called neurofibrillary tangles (NFT). Tangles are linked to the late stage of the pathology, where symptoms to a large degree are non-reversible whereas plaques build up occurs earlier in the disease and primarily before the onset of cognitive symptoms^2^. Future therapeutic approaches are aiming for early treatment, even presymptomatic, to effectively fight the disease progression. For this reason, it is important to study how the disease progresses before the appearance of the first plaques.

Over the last decade, the important role of the innate immune system in development of AD pathology has been well-established^3^. In relation to the innate-immune system, the microglial cells are crucial for the homeostatic clearance of Aβ, synaptic formation modulation and innate immune response, which all are suggested to be affected in the AD disease pathogenesis^4^. The inflammatory response has received special attention in AD, based on recent genome-wide association studies^5^. The inflammatory response elicited in AD by microglial cells has been linked to their activation by different Aβ species^6–8^. This chronic activation of microglial cells and the proinflammatory environment created due to microglial activity is suggested to be decisive for AD pathogenesis. It is plausible that early microglial activation can be one of the first pathological processes involved in the initiation of the disease due to the capacity of lower molecular Aβ species to activate microglial cells *in vitro*^9^. These Aβ molecules can be found in the extracellular environment, before the typical plaque deposition and by that activate/alter microglial function. This process can potentially initiate and drive the progression of the disease; not only by inducing proinflammatory microglial activation, but also by affecting the homeostatic function of microglia^10^, thereby affecting other cells in the brain such as neurons and astrocytes.

In view of recent failures of clinical drug trials when patients with established AD has been enrolled, the research community is searching for early pathological events important for the disease development. Therefore, elucidation of molecular pathways involved in the early phase in AD pathogenesis is necessary. In the present study, we aim to investigate alterations of the innate immune response in the microglial cell population in 5xFAD mouse model of AD. 5xFAD mouse model carries five different mutations to strongly upregulate the human amyloid metabolism, which is known to induce a strong microglial response^11^. We studied the progression of the pathology in the 5xFAD mice at 2, 6 and 10 weeks of age and related the readout to age-matched wild-type mice. By using mass spectrometry, together with immunohistochemistry and ELISA, we performed a proteomic analysis and identified the protein profile of microglial cells linked to the early stage of microglia activation in the pathology.

We found specific changes in isolated microglia before the plaque deposition at 10 weeks of age. Our study reveals a number of different pathways related with the innate immune system response, potentially involved in the progression of the disease, even before the first plaque deposition. Our results encourage a focus on early disease treatment and suggest further investigation of early microglial alteration in prodromal stages of AD.

## Materials and Methods

### Animals

We used male and female, same number of each, 5xFAD mice obtained originally from Jackson Laboratories, aged 2–10 weeks and weighting 14–20 g when starting the experiment. WT and 5xFAD were divided in groups depending on their age: 2 weeks, 6 weeks or 10 weeks. Each group contains 8 animals. The mice were bred and housed in standard laboratory cages with sawdust bedding and free access to water and food. The holding room had a 12:12 h light-dark cycle. The mice were randomly assigned to different experimental groups (5xFAD and WT) with no differences in body weight and age between the groups.

All animal experiments were performed in accordance with the animal research regulations (RD53/2013 and 2010/63/UE) in Sweden and European Union, and with the approval of the Committee of Animal Research at the University of Lund (Ethical permit number: M29-16).

### Genotyping

The genotypes of 5xFAD mice were determined using an integrated extraction and amplification kit (Extract-N-Amp™, Sigma-Aldrich). First, the samples were incubated at 94 °C for 5 mins, followed by 40 cycles with denaturation at 94 °C for 45 sec, annealing at 55 °C for 30 sec, and elongation at 72 °C for 1.5 mins. The following primers (CyberGene, Solna, Sweden) were used: For the 5xFAD the primers (5′ to 3′) used are listed below: APP Forward AGGACTGACCACTCGACCAG, APP Reverse CGGGGGTCTAGTTCTGCAT, PSN1 Forward AATAGAGAACGGCAGGAGCA, PSN1 Reverse GCCATGAGGGCACTAATCAT, WT APP Forward CTAGGCCACAGAATTGAAAGATCT, WTT APP Reverse GTAGGTGGAAATTCTAGCATCATCC, RD1, RD2 and RD3 AAGCTAGCTGCAGTAACGCCATTT,ACCTGCATGTGAACCCAGTATTCTATC, CTACAGCCCCTCTCCAAGGTTTATAG. The PCR products were separated by gel electrophoresis labelled with ethidium bromide and visualized using a CCD camera (SONY, Tokyo, Japan).

### Sequential Protein extraction

The protein extraction was performed in brain samples from 5xFAD at 2, 6 and 10 weeks and WT mice at 2 and 10 weeks. Mice were transcardially perfused under deep anesthesia with PBS, pH 7.4. Then, the mice were decapitated and the brains were removed. One brain hemisphere was snap frozen in dry ice and the other one was fixed in paraformaldehyde (4%) as described below. Soluble protein fractions were obtained from the whole cortex (mice) using sequential protein extraction. Fractions were obtained by homogenization of the cortex with a dounce homogenizer in the presence of PBS (1 mL/100 μg of tissue). After centrifugation for 1h at 43.000 rpm in special tubes for high-speed centrifugation from Beckman-Coulter (#357448, 1,5 ml tubes) the supernatants, were obtained and aliquoted and stored at −80 °C (S1 fraction). The pellets were extracted in RIPA buffer (Sigma-Aldrich, Germany), ultracentrifuge at 30.000 rpm and supernatants, S2 fractions (intracellular particulate proteins), were aliquoted and stored. PBS and RIPA solution were prepared using a protein inhibitor (Protein Inhibitor Cocktail, ThermoScientific) to prevent protein degradation and to inhibit the enzymatic activity of phosphatases (PhosphoStop, Roche).

### ELISA

A Mesoscale Discovery platform (MSD, from Rockville, Maryland, USA) was used to evaluate the cytokine levels by a proinflammatory reading plate for IFN-γ, IL-1β, IL-2, IL-4, IL-5, IL-6, IL-8, IL-10, IL-12 and TNF-α. To measure the cytokines in mouse brain soluble fractions, we pulled together 50 μg of the S1 and S2 fraction. Moreover, MSD plates were used to test the levels of human Aβ_40_ and Aβ_42_ in the soluble fraction of the brain extraction from WT and 5xFAD at different ages, 2, 6 and 10 weeks. Serial dilution of the soluble fraction was tested in order to obtain an accurate measure of the protein levels. The plates were developed using the 4× reading buffer diluted 1 time with distilled water and the plates were read using the QuickPlex Q120 reader from Mesoscale. The detection range of the different cytokines presented is the next (IL1β 1670-0,408 pg/mL: IL4, 1660-0,405 pg/mL; IL12, 32200-7,86; IL10, 3410-0,833). Other cytokines evaluated not included but not included in the results: IFN-γ (938-0,229 pg/mL), IL2 (2630-0,642 pg/mL), IL5 (967-0,236 pg/mL), IL6 (5720-1,40), KC/GRO (1980-0,483 pg/mL), TNF-α (627-0,153). Aβ_40_ detection range (15100-3,69 pg/mL), Aβ_42_ detection range (2280-0.557 pg/mL).

### Western blot

Western blotting was used to measure APP levels in brain extractions. The proteins were extracted from the cortex using PBS or RIPA buffer (Sigma-Aldrich, Germany) with proteinase and phosphatase inhibitors. The blots were run using pre-casted gels (4–20% from Bio-Rad) in TGS buffer (Bio-Rad). The proteins were transferred to nitrocellulose membranes (Bio-Rad) using the TransBlot turbo system from BioRad. The membranes were blocked for 1h with skim milk at 3% in PBS, then washed 3 × 10 mins in PBS and Tween-20 at 0,1%, after which they were incubated with primary and secondary antibodies diluted in PBS-Tween 20 at 0.1%. To develop the blot we use ECL Clarity (Bio-Rad) according to the manufacturer’s protocol and ChemiBlot XRS + system from Bio-Rad.

### Tissue processing and immunohistochemistry

Mice were transcardially perfused under deep anesthesia (5% isoflurane in oxygen) with PBS, pH 7.4. The brains were removed and fixed in 4% PFA for 24h, then, washed in PBS 3 times and then fixed in 30% sucrose solution for 3 days. Brains were cut at a 40 μm thickness in the sagittal plane on a microtome and collected in 6 series in cold PBS and 0.02% sodiumazide. Sections from control and 5xFAD mice were processed in parallel for light microscopy immunostaining using the same batches of reagents/solutions. Brief protocol: We rinsed 3 × 5 mins in PBS, pre-incubation with 10% NDS (normal donkey serum) in 0.25% Tween-20 in PBS (blocking solution) 1h in room temperature. Primary antibody staining (mouse 6E10 Covance 803002, 1:500; rabbit Iba1 Wako ref 019-19741, 1:500) was performed in 10% blocking solution (NDS, 0.25% Tween-20) and incubated overnight in room temperarture. The day after, we rinsed 3 × 5 mins in Tween-PBS (0.25%) and 1 × 5 mins in 10% blocking solution (NDS, 0.25% Tween-20). Secondary antibody staining (anti-mouse 555 Alexa Fluor and anti-rabbit 488 Alexa Fluor were used at 1:500) in 10% blocking solution for 2h in room temperature (dark conditions) was used followed by DAPI 1:1000 for 5 mins in 0.25% Tween X-100 in PBS and subsequent rinsing 3 × 5 mins PBS. Next, the sections were coverslipped with Pro-LongTM Diamond Antifade Mountant (Invitrogen) and dried in dark conditions in room temperature for 24 hours. For staining with Thioflavin-S (T1892, Sigma Aldrich) sections were first washed 3 × 5 mins in 0.25% Tween-20 in PBS and then incubated for 5 mins with 0.5% Thioflavin-S. Next, we washed sections 3 × 10 mins in 0.25% Tween-20 in PBS, mounted with Pro-LongTM Diamond Antifade Mountant (Invitrogen) and dried in dark conditions for 24 hours. Nikon Confocal A1+ microscope was used to take the pictures; 20× and 60× pictures where taken in the same area using NIS elements in-house software.

### Plaque quantification/microglial cells activation in the subiculum

Plaque load was defined as the number of Thioflavin-S positive Aβ plaques in the subiculum of 5xFAD mice. The number of labelled gal3/iba1 positive cells were quantified in the subiculum and CA1 pyramidal layer in the brains of 5xFAD. Images were acquired with a Nikon DS-5M high-resolution digital camera connected to a Nikon Eclipse 80i microscope. The camera settings were adjusted at the start of the experiment and maintained for uniformity. Digital 10× images from 2, 6 and 10 weeks old 5xFAD mice were used for plaque and microglial quantification. 6 sections/mouse; n = 4/age/genotype. Analysis of the fluorescent labelled structures was performed with Fiji ImageJ Software (W. Rasband, National Institutes of Health). The colour images for plaques were converted to binary images. Plaques and activated microglial cells were counted manually after setting the brightness threshold. Quantitative comparisons were carried out on sections processed at the same time with same batches of solutions. Pictures were retrieved from the same brain area in the different experimental groups.

### Isolation of microglial cells

Mice were transcardially perfused, under deep isoflurane anesthesia, with PBS pH 7.4 in order to remove any peripheral cells from the vasculature in the brain. Then, brains were removed from the cranium. Microglial cells from 5xFAD and WT brains were isolated from the right hemisphere using magnetic beads for CD11b surface marker (Miltenyi, Germany) along with isolation columns from Miltenyi Biomarkers. Briefly, the right hemisphere was dissected in small pieces and store in PBS. The suspension was spinned down and the tissue was processed using the Neuronal Tissue Dissociation Kit from Miltenyi (130-092-628, Germany) following the manufacturer’s protocol. The cell suspension was filtered using a 70 μM cell strainer. Next, we incubated our cell suspension with CD11b magnetic beads from Miltenyi for 30 mins at 4 °C and isolated the cells using the Miltenyi Isolation Columns. Once we isolated the cells, we added RIPA buffer (R0278, Sigma Aldrich) with phosphatase (04906837001, Roche) and proteinase inhibitors (04693159001, Roche) to avoid protein degradation.

### Flow cytometry

Microglia isolated by CD11b microbeads (Miltenyi Biotec) were analyzed for microglial cell surface antigens by flow cytometry. Briefly cells were incubated with anti-CD16/CD32 antibody (BD Bioscience, 1:100) to block Fc receptors. Samples were then incubated with anti-CD11b-APC (Biolegend, 1:800) and anti-CD45-PE (BD Bioscience; 1:400) to confirm purity of the microglial population. Gating was determined by proper negative isotype stained controls and compensation was made with single stainings. A viability staining, 7-aminoactinomycin D (7AAD)(BD Bioscience) was used to exclude dead cells. Flow cytometry was perfomed in a FACSArial III cytometer (BD Biosciences) and FACS Diva software (BD Biosciences). Ten thousand events were recorded and microglia were identified by CD11b^+^ and CD45^+^ expression^12^. Data analysis was made using FlowJo 10.3 software (Three Star, Inc). Confirmed the enrichment of microglial cells by flow cytometry using CD11b and CD45 microglial markers (Supp. Figure 1).

### Sample Preparation for proteomic analysis

Protein concentration was measured for each sample using Pierce™ BCA Protein Assay (Thermo Scientific, Rockford, USA) and the Benchmark Plus microplate reader (Bio-Rad Laboratories, Hercules, USA) with BSA solutions as standards. Aliquots of 1 or 4 μg of each protein extract sample were mixed into the pooled reference sample. Aliquots containing 10 μg of each experimental sample or reference sample were digested with trypsin using the filter-aided sample preparation (FASP) method^13^. Briefly, protein samples were reduced with 100 mM dithiothreitol at 60 °C for 30 mins, transferred on 30 kDa MWCO Nanosep centrifugal filters (Pall Life Sciences, Ann Arbor, USA), washed with 8 M urea solution and alkylated with 10 mM methyl methanethiosulfonate in 50 mM TEAB and 1% sodium deoxycholate. Digestion was performed in 50 mM TEAB, 1% sodium deoxycholate at 37 °C in two stages: the samples were incubated with 100 ng of Pierce MS-grade trypsin (Thermo Scientific, Rockford, USA) for 3 h, then 100 ng more of trypsin was added and the digestion was performed overnight. The peptides were collected by centrifugation labelled using TMT 10-plex isobaric mass tagging reagents (Thermo Scientific) according to the manufacturer’s instructions. The labelled samples from brain were mixed into corresponding sets, sodium deoxycholate was removed by acidification with 10% TFA.

The mixed labelled samples were subjected to the reversed-phase high pH fractionation on the AKTA chromatography system (GE Healthcare Life Sciences, Sweden) using the XBridge C18 3.5 μm, 3.0 × 150 mm column (Waters Corporation, Milford, USA) and 25 mins gradient from 7% to 40% solvent B at the flow rate of 0.4 ml/min; solvent A was 10 mM ammonium formate in water at pH 10.00, solvent B was 90% acetonitrile, 10% 10 mM ammonium formate in water at pH 10.00. The initial 31 fraction was combined into 15 pooled fractions in the order 2 + 17, 3 + 18, 4 + 19 etc. The pooled fractions were dried on Speedvac and reconstituted in 20 μl of 3% acetonitrile, 0.1% formic acid for analysis.

### LC-MS/MS Analysis

Each fraction was analyzed on Q Exactive mass spectrometer (Thermo Fisher Scientific, Bremen, Germany) interfaced with Thermo Easy-nLC 1200 nanoflow liquid chromatography system (Thermo Fisher Scientific, Odense, Denmark). Peptides were trapped on the C18 trap column (100 μm × 3 cm, particle size 3 μm) separated on the home-packed C18 analytical column (75 μm × 30 cm) packed with 3 μm Reprosil-Pur C18-AQ particles (Dr. Maisch, Germany) using the gradient from 6% to 32% B in 70 min, from 32% to 50% B in 5 min, from 50% to 100% B in 5 min; solvent A was 0.2% formic acid and solvent B was 80% acetonitrile, 0.2% formic acid. Precursor ion mass spectra were recorded at 70 000 resolution. The 10 most intense precursor ions were selected with the isolation window of 1.6, fragmented using HCD at stepped collision energy of 27, 35 and 47 and the MS^2^ spectra were recorded at a resolution 35 000. Charge states 2 to 6 were selected for fragmentation, dynamic exclusion was set to 30 s.

### Protein Evaluation

Data analysis was performed using Proteome Discoverer version 1.4 (Thermo Fisher Scientific, Waltham, USA). The protein database for *Mus musculus* (February 2017, 16854 sequences) was downloaded from Swissprot. Mascot 2.5.1 (Matrix Science) was used as a search engine with precursor mass tolerance of 10 ppm and fragment mass tolerance of 0.02 Da, one missed tryptic cleavage was accepted. Mono-oxidation on methionine was set as a variable modification, methylthiolation on cysteine and TMT-6 reagent modification on lysine and peptide N-terminus were set as a fixed modification. Percolator was used for the validation of identificated results; target false discovery rate of 1% was used as a threshold to filter confident peptide identifications.

Reporter ion intensities were quantified in MS3 spectra using Proteome Discoverer 1.4 at 0.003 Da mass tolerances with reporter absolute intensity threshold of 2000. The resulting ratios were normalized on the median protein value of 1.0 in each sample.

### Protein bioinformatics analysis

Proteins profile similarities were analysed by using Venny 2.1 (http://bioinfogp.cnb.csic.es/tools/venny/index.html) among the different analyzed groups. Then, Gene Ontology (GO) analysis and Panther pathways analysis database were used to analyse the data from 2 different approaches. Individual groups were analysed followed with group comparison. The first 200 of the most abundant proteins form each individual group (5xFAD 2, 6 and 10 weeks and WT 2 and 10 weeks) were used to evaluate the main pathways affected by EnrichR (Panther data base). Then, GO analysis was performed using STRAP software. After the GO analysis, the resulted top 20 upregulated and downregulated proteins selected from group comparison (5xFAD 2w vs 5xFAD 6w, 5xFAD 10w vs 5xFAD 6w, 5xFAD 10w vs WT12w and 5xFAD 2w vs WT 2w) were used to further analyze the main pathways involved. Briefly, to select the proteins for the GO analysis we did the following: Step 1; for each protein, calculate group means, fold changes and perform t-tests to get p-values, filter on fold change > 0 (to picks only up-regulated proteins) and sort on p-values. To get downregulated proteins we only change step 2; to filter on fold change < 0. The pathway analysis was performed using EnrichR (Panther database for pathways). EnrichR implements four scores to report enrichment results: p-value, q-value, rank (Z-score), and combined score.

The p-value is computed using a standard statistical method used by most enrichment analysis tools: Fisher’s exact test or the hypergeometric test. This is a binomial proportion test that assumes a binomial distribution and independence for probability of any gene belonging to any set.

The q-value is an adjusted p-value using the Benjamini-Hochberg method for correction for multiple hypotheses testing. The rank score or z-score is computed using a modification to Fisher’s exact test in which we compute a z-score for deviation from an expected rank.

Finally, the combined score is a combination of the p-value and z-score calculated by multiplying the two scores as follows:

$$c=\,{log}(p)\ast z$$

where c is the combined score, p is the p-value computed using Fisher’s exact test, and z is the z-score computed to assess the deviation from the expected rank.

PCA (principal analysis component) analysis was performed using Ingenuity Pathway Analysis (IPA) from Qiagen. PCA is a quality controls analysis and it was performed in order to detect outliers and to evaluate the variability within each group.

### Statistical analysis

The differences between experimental groups were analysed with one-way ANOVA with Tukey’s post hoc test or two-way ANOVA with Bonferroni post hoc test correction. P < 0.05 was considered as statistically significant. We used the statistical software GraphPad PRISM 7.0 (San Diego, CA, USA). Data is represented as mean ± S.D.

## Results

### Characterization of the disease progression in 5xFAD mice

**Figure 1:**
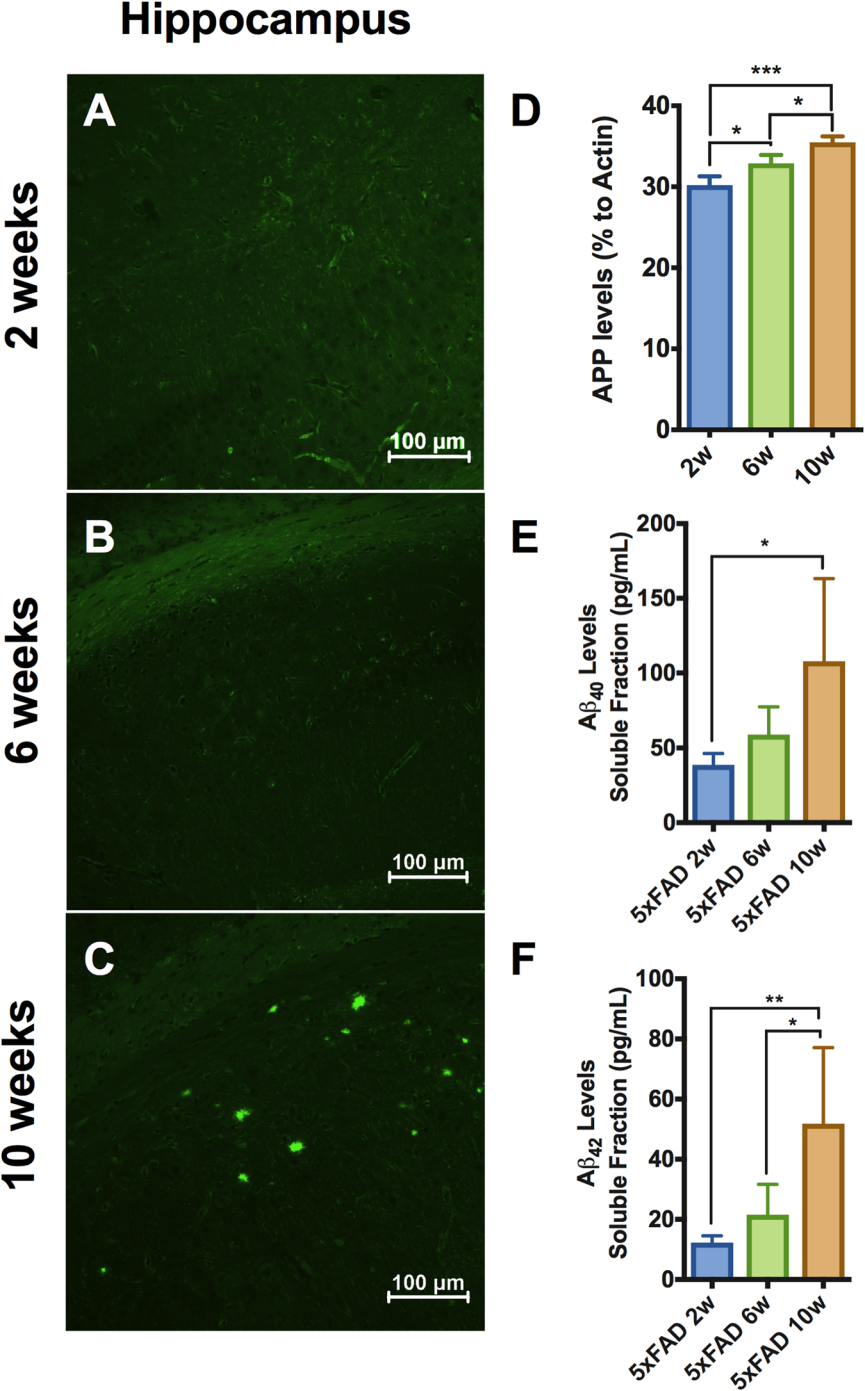
Characterization of AD brain pathology in 5xFAD mice. (**A**–**C**) Thioflavin-S staining on 5xFAD mice at 2 (**A**), 6 (**B**) and 10 (**C**) weeks. Only in 5xFAD mice at 10 weeks amyloid beta aggregates in the subiculum were present. n = 4 per group. (**D**) Human APP levels measured by western blot showed a significant increased in a time-dependent fashion from 2 weeks to 10 weeks in 5xFAD mice. (**E**,** F**) Aβ40 and Aβ42 levels were measured in brain homogenates soluble fraction by ELISA. (**E**) Aβ40 levels were significantly increased at 10 weeks compared to 2 weeks in 5xFAD mice. (**F**) Aβ42 levels were significantly increased in a time-dependent fashion in 5xFAD mice. n = 4 per group; *p < 0,05; **p < 0,005; One-Way ANOVA. Mean ± S.D.

We evaluated the age-dependent plaque deposition in hippocampus, the APP production and the brain extracellular Aβ levels in the 5xFAD mice to confirm the age-dependent Aβ production as part of the pathology. First, we stained our sections with thioflavin-S to visualize fibrillar plaques to verify the deposition of Aβ plaques. Thioflavin-S positive aggregates were found only in 10 week-old 5xFAD mice in the subiculum (Fig. 1C) but could not be detected at younger ages, 2 and 6 weeks (Fig. 1A, B) or in the WT mice (data not shown), which is in line with previous reports studying Aβ plaques in the 5xFAD mice^11,14^. Then, we used western blot to study the protein levels of the human amyloid precursor protein (APP) by using 6E10 antibody recognizing amino acid residues 1–16 in Aβ (Fig. 1D). We found a significant age-dependent increase of APP in the brain of 5xFAD mice from 2, 6 to 10 weeks of age (Fig. 1D). As expected, human APP was not detectable in WT brains (data not shown). Next, we evaluated by ELISA the human Aβ_40_ and Aβ_42_ levels in the soluble fraction from brain homogenates in the 5xFAD mice. We found a significant time-dependent increase in soluble Aβ_40_ and Aβ_42_ levels from 2 to 10 weeks of age (Fig. 1E, F). Previous studies have shown the ability of the soluble fraction to activate microglia^15,16^. Aβ_40_ or Aβ_42_, was not detected in WT mice (data not shown).

**Figure 2:**
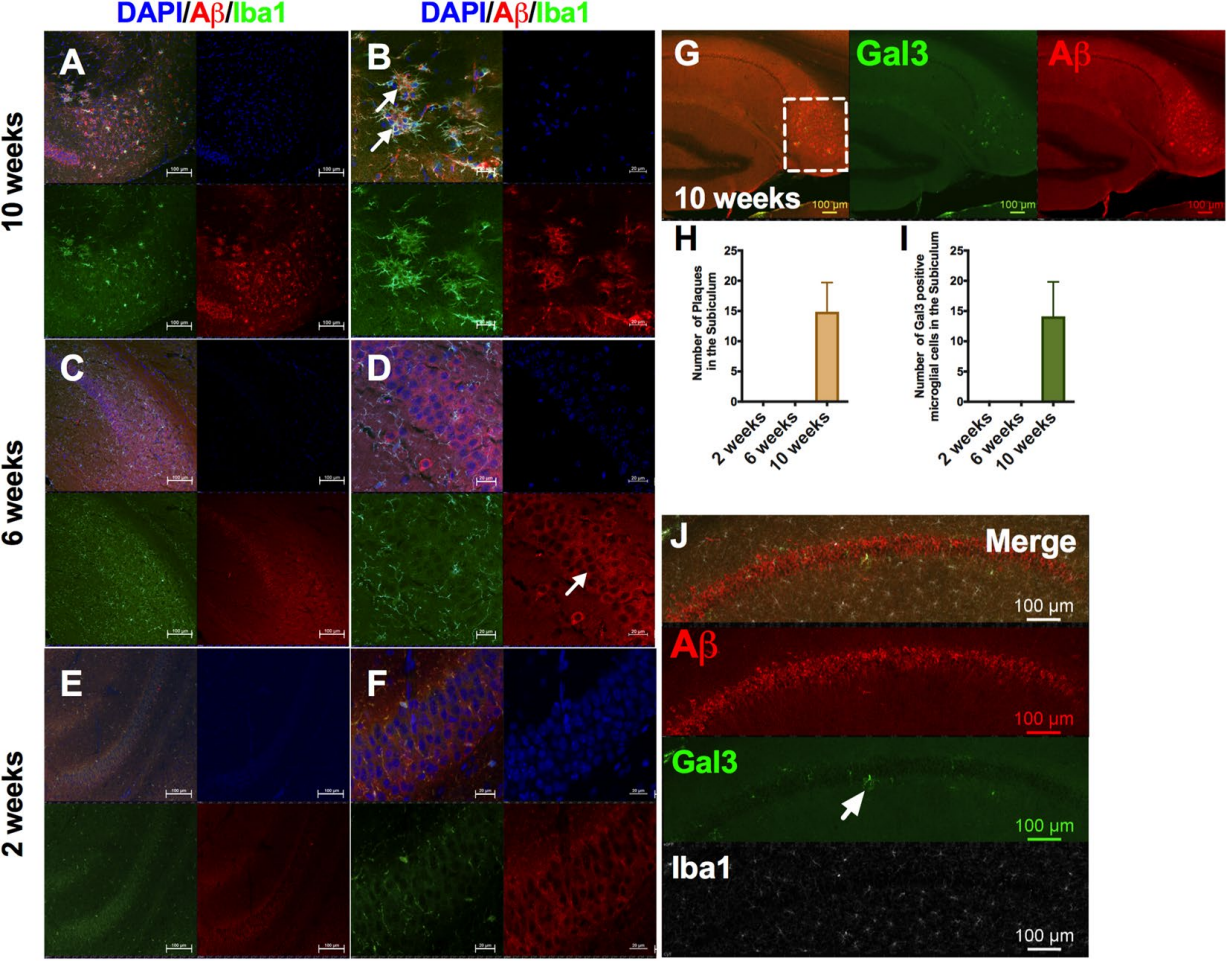
First plaque deposits appear at 10 weeks in 5xFAD mice in the subiculum subregion of hippocampus. Evaluation of the plaque deposition in 5xFAD from 2 to 10 weeks (**A**–**F**). (**A**,** B**) The first plaque deposits were present at 10 weeks. These plaque deposits were markedly surrounded by Iba1 positive cells (Details in B). Arrows indicated plaque deposits and Iba1 positive cells besides APP positive neurons (**B**). (**C**–**F**) At 2 and 6 weeks the plaque deposits were undetectable. At 6 weeks the APP positive neurons (white arrow in **D**) were more evident compared to 2 weeks (**E**,** F**). (**G**) Amyloid plaque depostis were found in the subiculum at 10 weeks. The squares indicates the area analyzed in the subiculum. (**H**) Aβ plaques quantification in the subiculum of 5xFAD mice from 2 to 10 weeks. (**I**) Gal3 positive microglial cells in the subiculum of 5xFAD from 2 to 10 weeks. Gal3 positive cells were Iba1 positive. (**J**) Gal3 positive microglial cells were found in the molecular layer in the hippocampus around APP positive cells at 6 weeks. DAPI: blue; 6E10 (Aβ): red; Iba1: Green. n = 3–4 per group.

To further confirm the plaque deposition, we stained our brain sections from 5xFAD mice with human anti-beta amyloid (6E10) antibody together with the microglial marker Iba1 (ionized calcium binding adaptor molecule 1) to evaluate the presence of microglial cells around plaques, a characteristic hallmark of the plaque deposition (Fig. 2). In line with our thioflavin-S results, we were able to confirm plaque deposits at 10 weeks in our 5xFAD mice in the subiculum (Fig. 2A, B, G and H), but not before that time-point in 5xFAD mice (6 and 2 weeks, Fig. 2C–F) or in WT mice (data not shown). Importantly, at 10 weeks we found the typical plaque-associated microglial cells (Iba1+) around the plaques (Fig. 2B). Then, we searched for activated microglial cells using galectin-3, which has been associated to proinflammatory microglial cells around Aβ plaque in 5xFAD brains^17^, and which we have found to be expressed in strongly activated microglial cells^17,18^ as a TLR4 ligand^19^. Galectin-3 positive cells could only be detected at 10 weeks and then in association with the plaque in subiculum (Fig. 2G and I). Interestingly, already at 6 weeks of age we found galectin-3 positive microglial cells in the CA1 pyramidal layer of the hippocampus around APP expressing neurons (Fig. 2J). This potential microglial activation at 6 weeks in the hippocampus might be related to the soluble Aβ released by APP positive neurons^7,15^. This finding is in line with our hypothesis about microglial activation before plaque deposition due to neuronal release of Aβ soluble molecules.

### Altered inflammatory cytokines in 5xFAD before plaque formation

**Figure 3:**
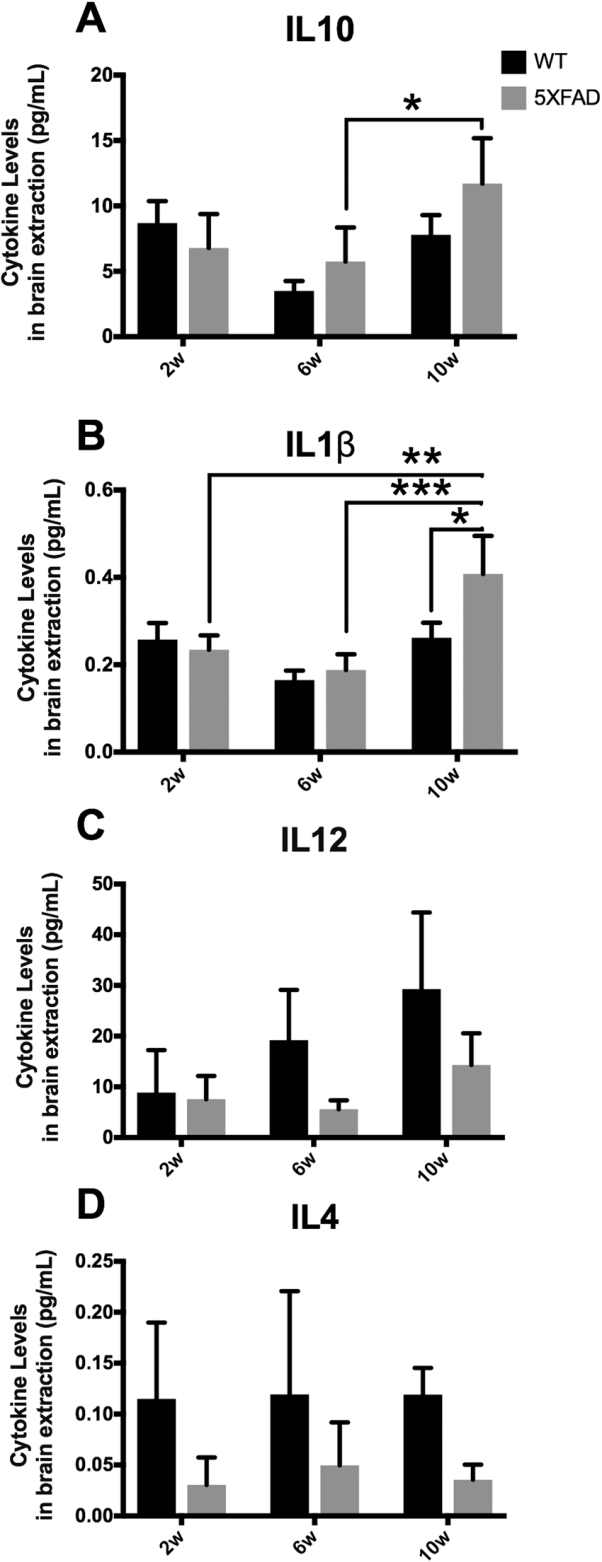
Altered cytokine pattern in brain homogenates from 5xFAD mice. ELISA evaluation of inflammatory cytokines from brain homogenates. (**A**) IL10, differences. (**B**) IL12, differences. (**C**) IL1β, differences. (**D**) IL4, differences. 5xFAD mice n = 5 for each 5xFAD group; WT n = 3 for each group. Mean ± S.D. mean. *p < 0,05; **p < 0,005; ***p < 0.001. Two-way ANOVA and One-way ANOVA with Tukey’s post correction for multiple comparisons between groups.

The presence of soluble Aβ_40_ and Aβ _42_ at early time points in the 5xFAD mice (2 and 6 week, Fig. 1E, F), despite absence of plaque, spurs the question if we still can detect early inflammatory changes. When analysing the brain tissue levels of the anti-inflammatory cytokine IL10 we found a significant time-dependent difference (Two-way ANOVA, p > 0.01) with a significantly upregulated by 41,6% at 10 weeks compared to 6 weeks in 5xFAD mice (Fig. 3A, p > 0.05). Similar results were obtained for the proinflammatory cytokine IL1β (Fig. 3B). Two-way ANOVA analysis showed a significant effect of both time and genotype (p > 0.001). Post hoc analysis revealed IL1β to be significantly upregulated with 60% at 10 weeks in 5xFAD mice compared to control mice, as well as 6 and 10 weeks in 5xFAD where it was increased by 54%, and compared to 2 weeks increased by 42,4% (Fig. 3B). The elevated levels of IL10 and IL1β at 10 weeks, the time point where plaques appears surrounded by activated microglial cells, could indicate that these two cytokines are related to the immune reactions elicited by the Aβ plaque. Next, we studied the cytokine IL12 and found significant time- and genotype-dependent differences (p > 0.05, two-way ANOVA). However, we failed to find any significant differences in post hoc analyses (Fig. 3C).

The last cytokine we found to be significantly altered between 5xFAD and WT, among all the pro- and anti-inflammatory cytokines measured (see material and methods), was the anti-inflammatory cytokine IL4 (Fig. 3D). We found IL4 to be downregulated in the brain of 5xFAD mice at all time points together compared to control animals (Fig. 3D, two-way ANOVA, p > 0.05), albeit independent time point differences were not significant in posthoc analysis (one-way ANOVA). The apparent early impact of IL4 level at 2 weeks in the 5xFAD mice suggest that APP expression and/or Aβ products can, at a very early age, alter IL4 levels in the brain.

### Bioinformatics analysis showed inflammatory pathways upregulated in 5xFAD microglial cells before plaque deposition

We used mass spectrometry in order to study protein alterations in microglia, qualitatively and quantitatively, in the WT and 5xFAD at 2 weeks and 6 weeks of age, before the formation of Aβ plaque, and at 10 weeks. Microglial cells were isolated from brains using CD11b magnetics beads (MACS).

**Figure 4:**
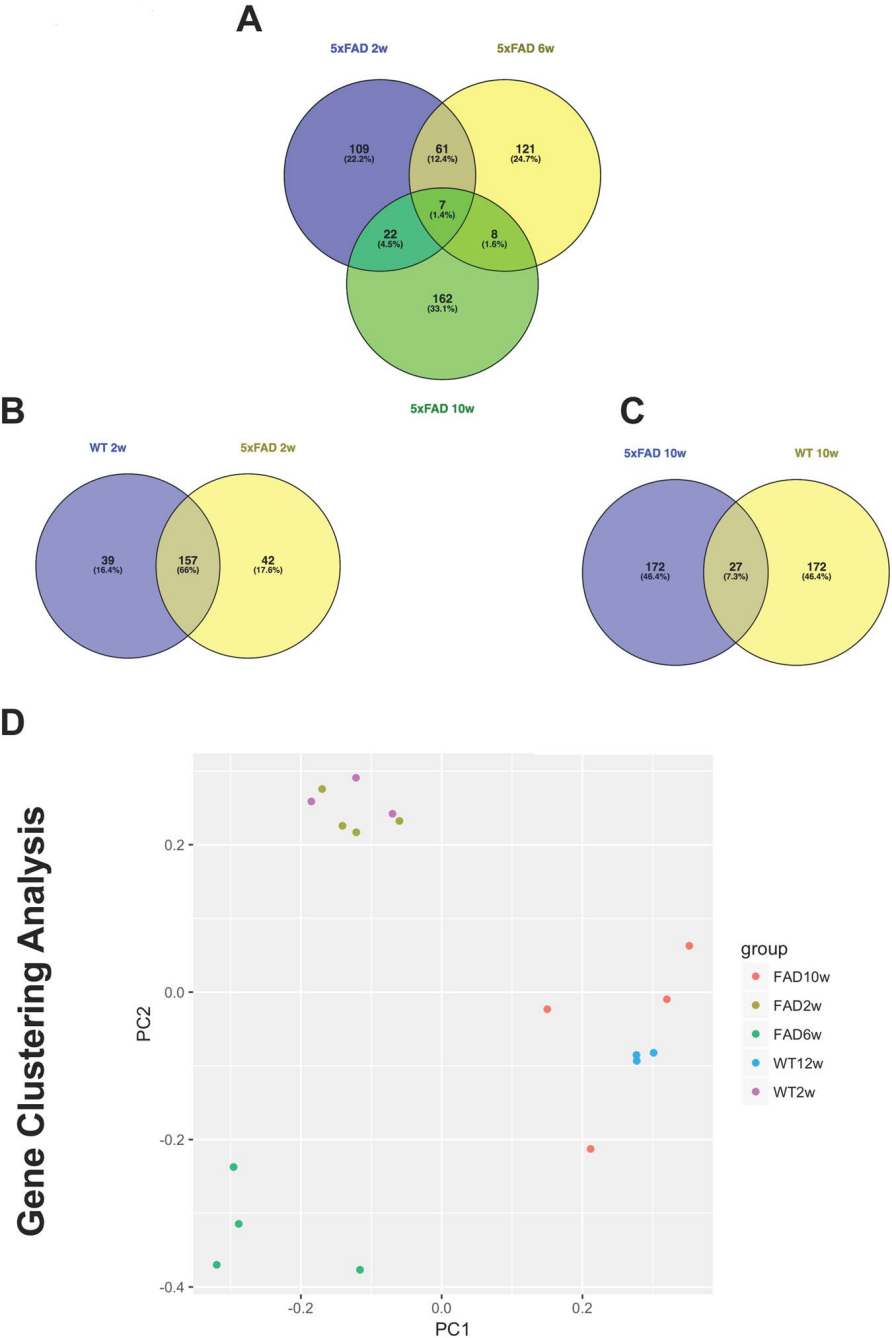
Protein profiles and PCA cluster analysis. (**A**) Comparison of the protein identity among the 200 most expressed proteins in 5xFAD. Among 2 and 6 weeks only 12.4% of proteins were the same. Among 6 and 10 weeks only 1.4% were the same. Among 2 and 10 weeks just 4.5% were the same. (**B**) Comparison between 2 weeks 5xFAD and 2 weeks WT mice results in 66% similarities in protein identity. (**C**) Comparison between 10 weeks 5xFAD and 10 weeks WT mice results in 7.3% similarities in protein identity. (**D**) Proteins cluster analysis (PCA). Proteins from the same group present a similar cluster. Analysis was performed using Ingenuity Pathways Analysis (IPA) from Quiagen.

Using a bioinformatic approach to understand the altered molecular and cellular processes, we analysed the 200 most abundant proteins at different time points and in the experimental groups. The protein profiles of 2 and 6 weeks 5xFAD microglia shared only 12,4% of the proteins (Fig. 4, analysed by software Veny 2.1 by CSIC). This difference was even larger when comparing 5xFAD at 6 and 10 weeks where only 1,6% of the proteins were the same, which could be related to the development of the Aβ plaque pathology. Surprisingly, the similarity between 2 and 10 weeks were larger with 4,5% similarity in the most 200 abundant proteins (Fig. 4A) which could indicate that early developmental programs at 2 weeks are also active at 10 weeks of age but in a pathological context. Comparing microglial proteins between 2 weeks, 6 weeks and 10 weeks, 5xFAD mice showed that 1,4% of the proteins were common in microglial cells from all of the time points studied (Fig. 4A). Interestingly, there were more proteins in common between 2 weeks old WT and 2 weeks old 5xFAD mice (66%) (Fig. 4B) than between 10 weeks WT and 10 weeks 5xFAD mice (7,3%) (Fig. 4C), indicating that microglial cells change their identity in response to the Aβ plaque deposition. Changes in microglial profile due to Aβ plaque formation have been shown in different mouse model and in human^20–22^. Moreover, as a data validation step, PCA analysis was performed to evaluate individual data point cluster in each group. Notably, no outliers were present and all groups formed clusters with some clusters overlapping each other (Fig. 4D).

Next, we analysed the protein profiles for the different experimental groups in terms of molecular pathways affected. The same 200 most abundant proteins were selected as before to analyse the main altered pathways in the 5xFAD mice at 2, 6 and 10 weeks of age. At 2 weeks in 5xFAD mice the main affected pathways were related to the RNA polymerase transcription and FAS ligand pathways (Table 1). Notably, already at 6 weeks we found inflammatory-related pathways affected. In fact, inflammatory pathways such as JAK/STAT, p38 MAPK and Interleukin pathways were among the most affected (Table 2). At 10 weeks in our 5xFAD mice, we found the vesicles trafficking as the most affect pathway followed by metabotropic and adrenergic regulation (Table 3), which is an interesting finding in this microglial cell population. The pathway analysis of the control groups (WT) did not show any inflammatory process affected at any time point (data not shown).

**Table 1:** Main affected pathways in 5xFAD at 2 weeks (top 200 most abundant proteins).

Index	Name	P-value	Adjusted p-value	Z-score	Combined score
1	General transcription by RNA polymerase I	0.0004042	0.01213	−1.25	5.51
2	FAS signaling pathway	0.003552	0.05329	−1.31	3.84
3	Transcription regulation by bZIP transcription factor	0.06544	0.3410	−1.05	1.13
4	Ubiquitin proteasome pathway	0.06819	0.3410	−0.83	0.89
5	CCKR signaling map ST	0.8093	0.8093	0.98	−0.21
6	Wnt signaling pathway	0.7668	0.7932	0.92	−0.21
7	PDGF signaling pathway	0.3066	0.4379	0.26	−0.21
8	Opioid prodynorphin pathway	0.1648	0.3709	0.23	−0.23
9	Heterotrimeric G-protein signaling pathway-Gi alpha and Gs alpha mediated pathway	0.3610	0.4709	0.35	−0.26
10	5HT4 type receptor mediated signaling	0.1479	0.3709	0.26	−0.26

**Table 2:** Main affected pathways in 5xFAD at 6 weeks (top 200 most abundant proteins).

Index	Name	P-value	Adjusted p-value	Z-score	Combined score
1	JAK/STAT signaling pathway	0.0003162	0.01138	−1.44	6.43
2	PDGF signaling pathway	0.005052	0.06063	−1.46	4.09
3	p38 MAPK pathway	0.003783	0.06063	−1.40	3.94
4	General transcription regulation	0.03085	0.1915	−0.65	1.07
5	Insulin/IGF pathway-mitogen activated protein kinase kinase/MAP kinase cascade	0.03292	0.1915	−0.64	1.05
6	FAS signaling pathway	0.03723	0.1915	−0.54	0.89
7	Transcription regulation by bZIP transcription factor	0.06429	0.2893	−0.69	0.86
8	General transcription by RNA polymerase I	0.009316	0.08384	−0.31	0.76
9	Ras Pathway	0.1480	0.3674	−0.54	0.54
10	Interleukin signaling pathway	0.2078	0.4280	−0.07	0.06

**Table 3:** Main affected pathways in 5xFAD at 10 weeks (top 200 most abundant proteins).

Index	Name	P-value	Adjusted p-value	Z-score	Combined score
1	Synaptic vesicle trafficking	0.000002697	0.00009360	−1.42	13.19
2	Metabotropic glutamate receptor group II pathway	0.00001080	0.0002267	−1.47	12.34
3	Beta3 adrenergic receptor signaling pathway	0.000002971	0.00009360	−1.12	10.42
4	Opioid prodynorphin pathway	0.00002609	0.0002348	−0.92	7.72
5	5HT4 type receptor mediated signaling pathway	0.00001576	0.0002348	−0.91	7.63
6	Opioid proopiomelanocortin	0.00002609	0.0002348	−0.85	7.07
7	Muscarinic acetylcholine receptor 2 and 4 signaling	0.00004056	0.0003194	−0.79	6.38
8	Muscarinic acetylcholine receptor 1 and 3 signaling	0.00005849	0.0004095	−0.76	5.93
9	Opioid proenkephalin	0.00002609	0.0002348	−0.65	5.44
10	Metabotropic glutamate receptor group III pathway	0.0001974	0.0009568	−0.39	2.68

**Figure 5:**
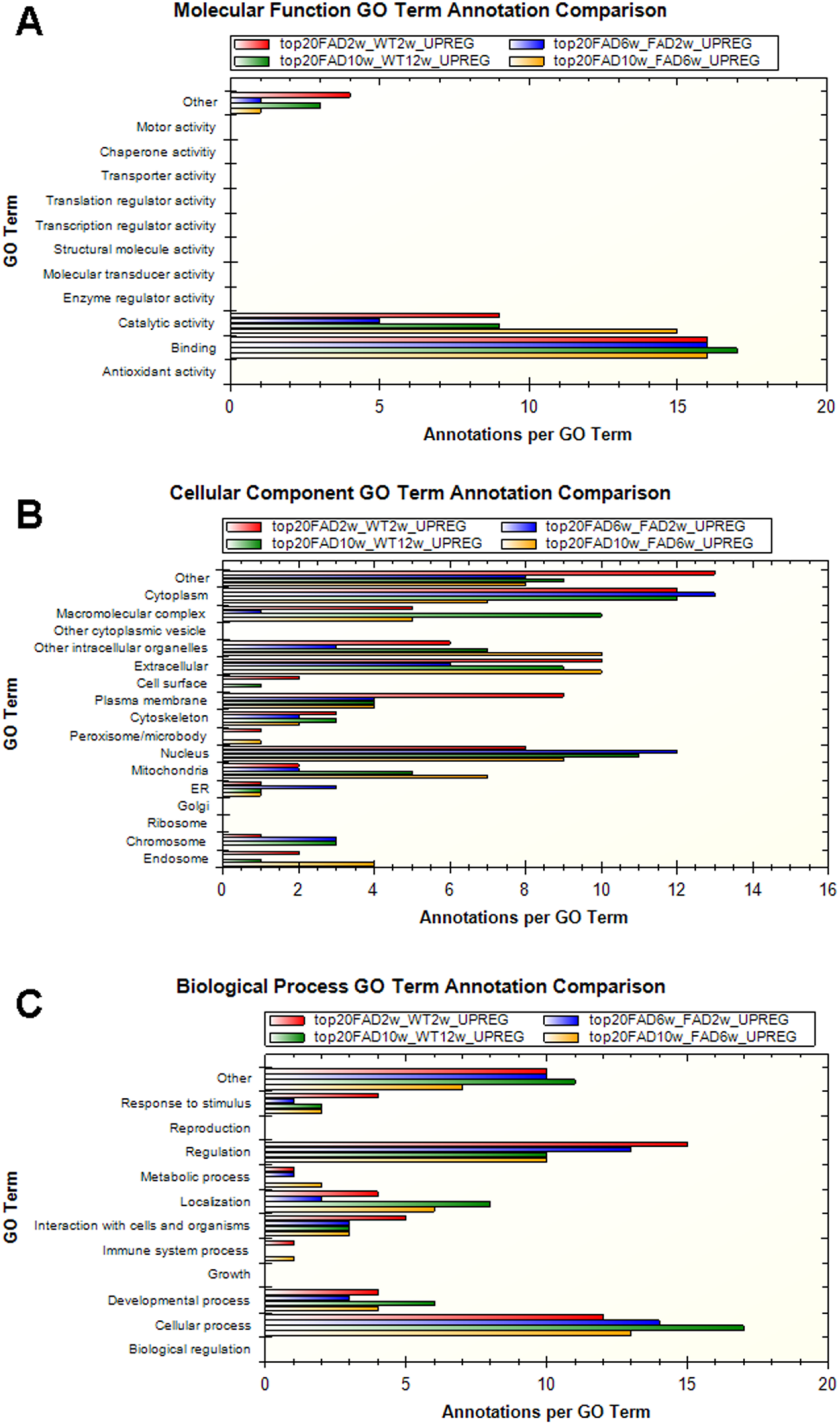
Gene Ontology Analysis. (**A**) Molecular function analysis on the top 20 upregulated proteins among different groups. (**B**) Cellular Component analysis on the top 20 upregulated proteins among different groups. (**C**) Biological Processes analysis on the top 20 upregulated proteins among different groups. Red, 5xFAD 2 weeks vs WT 2 weeks; Blue, 5xFAD 6 weeks vs 5xFAD 2 weeks; Green, 5xFAD 10 weeks vs WT 10 weeks; Yellow, 5xFAD 10 weeks vs 5xFAD 6 weeks. STRAP software was used to perform the GO analysis.

For the Gene Ontology (GO) analysis, we studied the molecular function, cellular component and biological process between different groups. We first investigated the very first age-related changes linked to AD pathogenesis by making a comparison between 5xFAD 2 weeks and 5xFAD 6 weeks. The molecular function was mostly affected in regards to the catalytic activity and the binding (Fig. 5A). Notably, both catalytic and binding activity were upregulated in a time-dependent manner in 5xFAD from 2 to 10 weeks and in comparison to WT (Fig. 5A) Next, we studied subcellular components related to the protein alterations in microglial cells. We found the endoplasmatic reticulum (ER), the cytoplasmatic compartment and the nucleus to be the main cellular components affected at 6 weeks compared to 2 weeks of age in the 5xFAD mice in relation to other affected cellular components (Fig. 5B). For the biological processes: cell regulation and cellular processes were clearly affected in 5xFAD at 6 weeks compared to 5xFAD at 2 weeks (Fig. 5C). Notably, the GO for the biological processes showed enrichment of the proteins related with immune system processes in the 5xFAD at 10 weeks compared to 5xFAD 6 weeks (Fig. 5C), in line with our hypothesis of inflammation development over time. The main cellular components altered at 10 vs 6 weeks in the 5xFAD microglial cells were the endosome and the mitochondria, in relation to other affected cellular components (Fig. 5B). Finally, we analysed the pathways affected for each compared group by using the top 20 proteins upregulated resulting from the GO analysis for both 5xFAD 10 and 6 weeks (Table 4) and 5xFAD 6 and 2 weeks (Table 5). Interestingly, the comparison between 6 and 2 weeks 5xFAD showed an increasing regulation of the FAS ligand pathways at 6 weeks old 5xFAD (Table 6B). The pathways analysis outcome between 10 weeks and 6 weeks old 5xFAD mice showed a clear upregulation of immune system related pathways such as interferon-gamma signalling pathways and a strong trend for altered B and T cell activation pathways and interleukin pathways in the 10 weeks old 5xFAD mice (p = 0.055, p = 0.071, respectively) which goes along with the alteration of cytokines we detected in 10-week old 5xFAD brains. Importantly, we found a strong trend in Alzheimer’s disease related-pathways such as the amyloid-secretase pathway to be upregulated at 10 weeks in microglial cells from the 5xFAD (p = 0.055, Table 6A).

**Table 4:** Gene Ontology Top 20 5xFAD 10 weeks upregulated proteins compared to 5xFAD 6 weeks.

Proteins	Name	Location
Ppp1r21	Protein phosphatase 1 regulatory subunit 21	2p16.3
Gtf3c5	General transcription factor IIIC subunit 5	9q34.13
Smchd1	Structural maintenance of chromosomes flexible hinge domain containing 1	18p11.32
Tdrd7	tudor domain containing 7	9q22.33
Sugp1	SURP and G-patch domain containing 1	19p13
Sh3glb1	SH3 domain containing GRB2 like, endophilin B1	1p22.3
Cherp	Calcium homeostasis endoplasmic reticulum protein	19p13.11
Fam129a	Family with sequence similarity 129 member A	1q25.3
Cenpv	Centromere protein V	17p11.2
Coasy	Coenzyme A synthase	17q21.2
Ndrg3	NDRG family member 3	20q11.23
Plek	pleckstrin	2p14
Prpf6	pre-mRNA processing factor 6	20q13.33
Fmnl3	formin like 3	12q13.12
Mad1l1	MAD1 mitotic arrest deficient like 1	7p22.3
Anxa7	annexin A7	10q22.2
Cmtr1	cap methyltransferase 1	6p21.2
Gvin1	GTPase, very large interferon inducible pseudogene 1	11p15.4
Parp2	Poly(ADP-ribose) polymerase 2	14q11.2
Gnl3	G protein nucleolar 3	3p21.1

**Table 5:** Gene Ontology Top 20 5xFAD 6 weeks upregulated proteins compared to 5xFAD 2 weeks.

Proteins	Name	Location
Mapk1	Mitogen-activated protein kinase 1	22q11.22
Cul3	cullin 3	2q36.2
Rragc	Ras related GTP binding C	1p34.3
Mfge8	Milk fat globule-EGF factor 8 protein	15q26.1
Acad8	Acyl-CoA dehydrogenase family member 8	11q25
Acads	Acyl-CoA dehydrogenase, C-2 to C-3 short chain	12q24.31
Colgalt1	Collagen beta(1-O)galactosyltransferase 1	19p13.11
Dak	triokinase and FMN cyclase	11q12.2
Cobll1	Cordon-bleu WH2 repeat protein like 1	2q24.3
Uba5	Ubiquitin like modifier activating enzyme 5	3q22.1
Scamp2	Secretory carrier membrane protein 2	15q24.1
Rab5c	RAB5C, member RAS oncogene family	17q21.2
Mccc2	methylcrotonoyl-CoA carboxylase 2	5q13.2
Eci2	enoyl-CoA delta isomerase 2	6p25.2
Clybl	citrate lyase beta like	13q32.3
Ttr	transthyretin	18q12.1
Rab4a	RAB4A, member RAS oncogene family	1q42.13
Wdr7	WD repeat domain 7	18q21.31
Ppt1	palmitoyl-protein thioesterase 1	1p34.2
Clpp	caseinolytic mitochondrial matrix peptidase proteolytic subunit	19p13.3

**Table 6:** Main innate-related pathways resulted from top 20 most upregulated proteins from the Gene Ontology.

Pathway 5xFAD 10w vs 6w	P-value	Adjusted p-value	Z-score	Combined score
Interferon-gamma signaling pathway	0.02764	0.1262	−1.45	3.01
Insulin/IGF pathway-mitogen activated protein kinase kinase/MAP kinase cascade	0.02862	0.1262	−1.25	2.59
B cell activation	0.05551	0.1262	−1.15	2.38
Alzheimer disease-amyloid secretase pathway	0.05456	0.1262	−1.13	2.34
Ras Pathway	0.06682	0.1262	−1.05	2.18
T cell activation	0.07056	0.1262	−0.90	1.87
Interleukin signaling pathway	0.08261	0.1262	−0.65	1.34
**Pathways 5xFAD 6w vs 2w**	**P-value**	**Adjusted p-value**	**Z-score**	**Combined score**
FAS signaling pathway	0.03056	0.03056	−1.46	5.10

## Discussion

In this study, we investigated if altered inflammatory pattern in the commonly used AD mouse model 5xFAD could be found before plaque deposition. We studied inflammatory alterations related to microglial cells and we found a clear inflammatory activation in microglial cells isolated before Aβ plaque deposition with altered cytokine levels in the 5xFAD brain and upregulation of proteins related to JAK-STAT, MAPK and Interleukins pathways along with a significant increase in the soluble Aβ levels. Moreover, at 10 weeks of age, the inflammatory pathways highlighted were linked to vesicles metabolism, which has been recently related to amyloid toxicity and plaque formation^23^. Moreover, at 6 and 10 weeks we found gal3 positive microglial cells around APP neurons and Aβ, respectively. Gal3 has been shown to be involved in the proinflammatory activation of microglial cells and it is expressed in microglial cells around Aβ plaques in 5xFAD mice^17,19^. The data obtained in this study demonstrate the role of microglial cells in the inflammatory response and the dynamics of the response from pre-plaque stages to the appearance of the first plaques.

This early aspect of microglial activation has received a lot of attention recently, and there are many studies trying to elucidate the dynamics of the inflammatory response in the context of AD. At present, the majority of studies have used APP-PS1 models in adult or aged mice, or even APP rats^24^, with a wide range of interesting approaches, from studying complement system and synapsis loss^20^ to autophagy in microglial cells^25^. For instance, López-Gonzaléz *et al*., highlighted the upregulation of proinflammatory related genes in APP-PS1 mice in adult and aged mice (from 3 to 12 months)^26^. Our approach is focusing on very early alterations before the typical disease manifestation, from 2 weeks to 10 weeks, in the 5xFAD mouse model, using mass spectrometry to explore the protein expression in isolated microglial cells.

In the present study, we first determined when and where the Aβ deposition takes place. By using thioflavin-S and Aβ staining, we found Aβ deposits at 10 weeks restricted to the subiculum, a medial subregion of the hippocampus (Figs 1C and 2A). We found soluble levels of Aβ_40_ and Aβ_42_ to be increased before the plaque deposition (Fig. 1E,F).

Microglial activation is one of the key events in AD progression. We evaluated the microglial activation based on gal3 expression, a marker of proinflammatory microglial activity^19^, and we found a clear microglial activation in microglial cells at 10 weeks around Aβ plaques (Fig. 2G) in the subiculum and sparsely around APP neurons at 6 weeks. (Fig. 2J), before any plaque deposit can be found in the brain. The gal3 immunoreactivity at 6 weeks of age in the molecular layer of hippocampus is especially interesting due to the role of gal3 positive microglial cells in the inflammatory response. Previously, we have found a detrimental role of gal3 in microglial activation in model of α-synuclein-induced microglial activation related to Parkinson’s disease pathogenesis^18^. In line with our inflammatory hypothesis, our present data demonstrate the presence of activated microglial cells in the hippocampus of 5xFAD in time points before plaque deposits.

Over the last decade, a large interest in the contribution of the innate immune system and its role in AD have been incurred. Especially, the microglial cells, have received a lot of attention. Understanding how the inflammatory response contributes to AD can be important to halt the progression of the disease. Hence, we aim to elucidate early inflammatory events related to Aβ immune reactions.

Here, we show an altered cytokine pattern in 5xFAD mice compared to control mice. In our study, protein levels of the cytokines IL10, IL12, IL1β and IL4 (Fig. 3) were altered in the early phase of the pathology in 5xFAD mice. When we further analysed cytokine levels, we found IL10 and IL1β to be significantly elevated in 5xFAD mice first at 10 weeks whereas IL12 showed a strong trend towards reduction starting at 6 weeks in 5xFAD mice compared to control animals, *i.e*. before the formation of plaque, albeit not significant. Interestingly, the anti-inflammatory cytokine IL-4 was overall reduced in the brain of 5xFAD mice at 2, 6 and 10 weeks of age.

IL10 is an anti-inflammatory cytokine known to cease a proinflammatory response^27^, presenting a significant up regulation from 6 weeks (pre-plaque stage) to 10 weeks of age in the brains of 5xFAD mice (Fig. 3A), suggesting IL10 to be related to the pathology starting at the time/or due to plaque formation. The function of IL10 and its effects on AD pathogenesis has been studied recently. For instance, Guillot-Sestier and colleagues found IL10 deficiency to reduce the disease progression in APP-PS1 crossed with IL10 knockout mice^28^. They found a preserved synaptic integrity and altered regulation of innate immune genes with increased Aβ phagocytosis. Using a different approach, the role of IL10 was investigated and the outcome basically the same in a work where Chakrabarty *et al*., demonstrated how the up regulation of IL10, performed by the injection of an AAV2/1*-IL-10 expressing viral vector* increased the amyloid deposition and worsen the cognitive behaviour in an AD mouse model^29^. Taken together, these reports suggest IL10 to be involved in AD pathology, in line with our IL10 cytokine increase, at the time of plaque formation. The overall role of IL10 in the human brain for disease outcome and association to Aβ microgliosis is still unresolved.

Further, we found the pivotal proinflammatory cytokine IL1β to be elevated in the brains of 5xFAD mice at the time of plaque deposition at 10 weeks of age (Fig. 3B). IL1β has also been studied in experimental AD models, APPswe/PS-1dE9, where overexpression of IL1β resulted in altered plaque pathology^30^. IL1β has also been genetically related to AD, where polymorphism in the IL1β gene can be related to AD progression and sleep disorders^31^. Moreover, elevated levels of IL1β were found in the cerebrospinal fluid of AD patients indicating clinical relevance for mechanisms involving this cytokine in AD development.

Another cytokine that we found a genotype-related difference (overall effect, 2-way ANOVA) was IL12 (Fig. 3C). IL12 is suggested to be involved in the progression of AD pathology. In fact, IL12 inhibition reduces AD pathology in APP/PS1 mice by reducing plaque formation and cognitive deficits after intra-cerebroventricular injection of antibodies inhibiting IL12/IL23 signaling^32^. Clinically, elevated levels of IL12 subunit p40 has been found in CSF from AD patients^32^. However, a dual role of IL12 in the inflammatory response has been described, by regulation of IL10 by the activation of Th1 cells^33,34^, showing the complex immune interplay involving IL12. Finally, we found one of the principal anti-inflammatory cytokine, IL4, to be reduced in 5xFAD mice compared to WT (Fig. 4D, overall effect, 2-way ANOVA). This downregulation of IL4 suggests that this cytokine is sensitive in early stages of the pathology. IL4 is considered an anti-inflammatory cytokine and may play a role in microglial activation^35^.

To further elucidate the microglial phenotype, we isolated microglial cells from 5xFAD and WT brains and performed bioinformatic analysis of microglia protein profile generated by mass spectrometry. Mass spectrometry allowed us to precisely quantify and identify the proteins expressed in microglial cells from 5xFAD and WT mice. It is important to mention that at 10 weeks, the Aβ plaque is only present in the subiculum and it is only a very limited number of cells directly in contact with the plaques compared to the number of microglial cells isolated from the whole cortex and hippocampus (more than 250.000 microglial cells/brain analysed).

At week 2, we found processes linked to RNA transcription by RNA polymerase I and FAS signaling pathway affected (Table 1). FAS ligand signalling is an innate immune pathway related to apoptotic mechanisms and inflammatory response. For instance, the highlight of the apoptotic pathways can be linked to brain development connected to TNF-α expression^36^.

Remarkably, at 6 weeks, we detected altered innate-immune related pathways in our microglial cells isolated from the 5xFAD mice (Table 2). These pathways were: JAK/STAT, p38 MAPK and Interleukin related pathways. All of them are directly involved in the inflammatory response elicited by microglial cells. For instance, p38 MAPK protein has been linked with microglial response by directly promoting the expression of inflammatory cytokine such as TNF-α, IL6 or IL-1β via TLR’s ligands or in the presence of Aβ^37^. Moreover, p38 MAPK has been considered a suitable target for AD^38,39^. The role of interleukins in the inflammatory response has been widely studied and it’s implication in AD progression discussed above^32,40–43^. Remarkably, at 10 weeks, we found synaptic vesicle trafficking as the most altered pathway in our analysis. We did not found the previous proinflammatory pathways up regulated among the main pathways affected at 10 weeks compared to the pathways described at 6 weeks. However, individual proinflammatory proteins found at 6 weeks ranked higher at 10 weeks (see supplementary excel file). The prevalence of vesicles trafficking pathways over proinflammatory pathways at 10 weeks is likely related with the beginning of the plaque formation and deposition, as we found in the subiculum. Furthermore, the synaptic vesicle trafficking has been linked to the inflammatory response^23^. This data highlight the sensitivity of our approach, being able to address minor and important changes in the dynamic of the innate immune response. For instance, vesicles-like structures, *i.e*. exosomes and microvesicles are suggested to be involved in brain disorders such as Parkinson’s disease disease and AD^23,44,45^. In line with the role of microvesicles in AD, a recent paper published by Verderio’s lab, shown that microvesicles released by microglial cells are linked to Aβ aggregation and neurotoxicity^23^.

To further analyse our proteomic data, we performed a GO analysis to establish the main molecular function, cellular component and biological processes affected. Regarding the molecular function, the most striking difference was found when comparing 5xFAD 6 and 2 weeks of age (Fig. 5A). The catalytic activity was upregulated at 6 weeks compared to 2 weeks 5xFAD mice. Among the proteins with a catalytic activity mainly upregulated in our analysis, we found protein phosphatase 1 regulatory subunit 21 (Ppp1r21) and GTPase protein, very large interferon inducible 1 (Gvin1) (Table 4). Phosphatases such as serine/threonine and tyrosine have been linked with AD pathology^46^. Gvin1, which is related with inflammatory response is upregulated in our 5xFAD in line with other studies^11^.

Notably, the biological processes affected between 5xFAD at 10 and 6 weeks of age pointed out significant differences in the innate immune response (Fig. 5C). In fact, proteins such as Mapk1, Rragc and Dak were among the top 20 upregulated (Table 5). Mapk1, mitogen-activated protein kinase 1, is a protein belonging to the MAPK protein family. MAPK proteins have been widely studied and linked to microglial activation and IL1β production in AD^47^. For instance, microglial p38α MAPK is a regulator of proinflammatory cytokine expression induced by TLR ligands and Aβ^37^.

The comparison from 5xFAD 6 weeks vs 2 weeks confirmed FAS ligand signalling pathway as the main up regulated pathway at 2 weeks of age (Table 5). Then, we made a comparison between 5xFAD at 10 and 6 weeks and we detected a number of different innate-immune related pathway affected such as: interferon gamma regulation, interleukins-related pathways and B and T cells activation pathways, which were upregulated in 5xFAD at 10 weeks compared to 6 weeks (Table 6). Our data demonstrate the implication of the innate immune response from 6 to 10 weeks based on the cytokine levels and especially due to the innate immune pathways upregulated at 6 weeks (MAPK p38 kinase, JAK-STAT or interleukin pathways) and 10 weeks (synaptic vesicles and metabolic alterations), as well as, microglial cells expressing gal3 around APP positive neurons in the hippocampus before plaque deposition. Future analysis of the proteomic of single cells, especially in relation to Aβ plaque, using CyTOF mass cytometry^48^ or single cell RNAseq^49^ can be useful to pin-point the detrimental signaling in microglia that contribute to the neuroinflammatory pathogenesis in AD.

## Conclusion

In this study, we describe microglial changes in the very early phase of AD pathogenesis before plaque deposition in the brain of 5xFAD mice. We believe this approach can be useful to better understand the cellular pathways involved in the early disease progression, before any clinical manifestation of the disease. By performing proteomic analysis, and using bioinformation analysis to interpret the data, we were able to describe the dynamics of the inflammatory response from 2 to 10 weeks in 5xFAD AD mouse model. First, we detected important inflammatory pathways up regulated at 6 weeks (MAPK, JAK/STAT and Interleukin pathways) and then, at 10 weeks of age, we found a shift in the microglial profile with a predominant action of vesicle trafficking. Moreover, using ELISA we were able to validate our mass spectrometry data analysis and found innate immune alterations in important neuroinflammatory cytokines, IL10 and IL1β related to the time point of plaque deposition at 10 weeks. We believe that therapeutic approaches, where early microglial alteration can be targeted, are crucial in future pre-clinical AD trials.

## Electronic supplementary material

Flow Cytometry

Related Manuscript File
