# Supplementary material for: Innate immune alterations are elicited in microglial cells before plaque deposition in the Alzheimer’s disease mouse model 5xFAD
Source: Sci Rep. 2018 Jan 24;8:1550. doi: 10.1038/s41598-018-19699-y (PMC5784016; doi:10.1038/s41598-018-19699-y)
Supplement: Supplementary file 1 — Flow Cytometry [file 41598_2018_19699_MOESM1_ESM.pdf]

**Innate immune alterations are elicited in microglial cells before plaque deposition in the Alzheimer's disease mouse model 5xFAD**

Antonio Boza-Serrano*[1], Yiyi Yang*[1], Agnes Paulus[1] and Tomas Deierborg[1]

Experimental Neuroinflammation Laboratory, Department of Experimental Medical Science, Lund University, Lund, Sweden.

Sölvegatan 19, BMC B11, 221 84 Lund, Sweden

Corresponding author: Antonio Boza-Serrano; antonio.boza_serrano@med.lu.se

Tomas Deierborg; tomas.deierborg@med.lu.se

*Equal contribution to the paper

**Keywords:** inflammation, brain, Alzheimer's disease, innate immune system and microglia.

**Supplementary Figure 1**

**Enrichment of microglia after CD11b microbeads isolation**

A) Bars show a relative comparison of microglia that were CD11b$^+$ and CD45$^+$ before and after CD11b microbeads isolation. There is a significant enrichment of microglia population after the isolation compared to before (Paired t-test, n = 4, $P < 0.05$, error bars with mean ± SEM).

(B-C) Representative contour plots of CD11b microbeads isolated cells stained with anti-CD11b-APC and anti-CD45-PE antibodies. Microglia were identified by double

positive for CD11b and CD45 staining. Only single and live cells were taken into consideration for the gating.

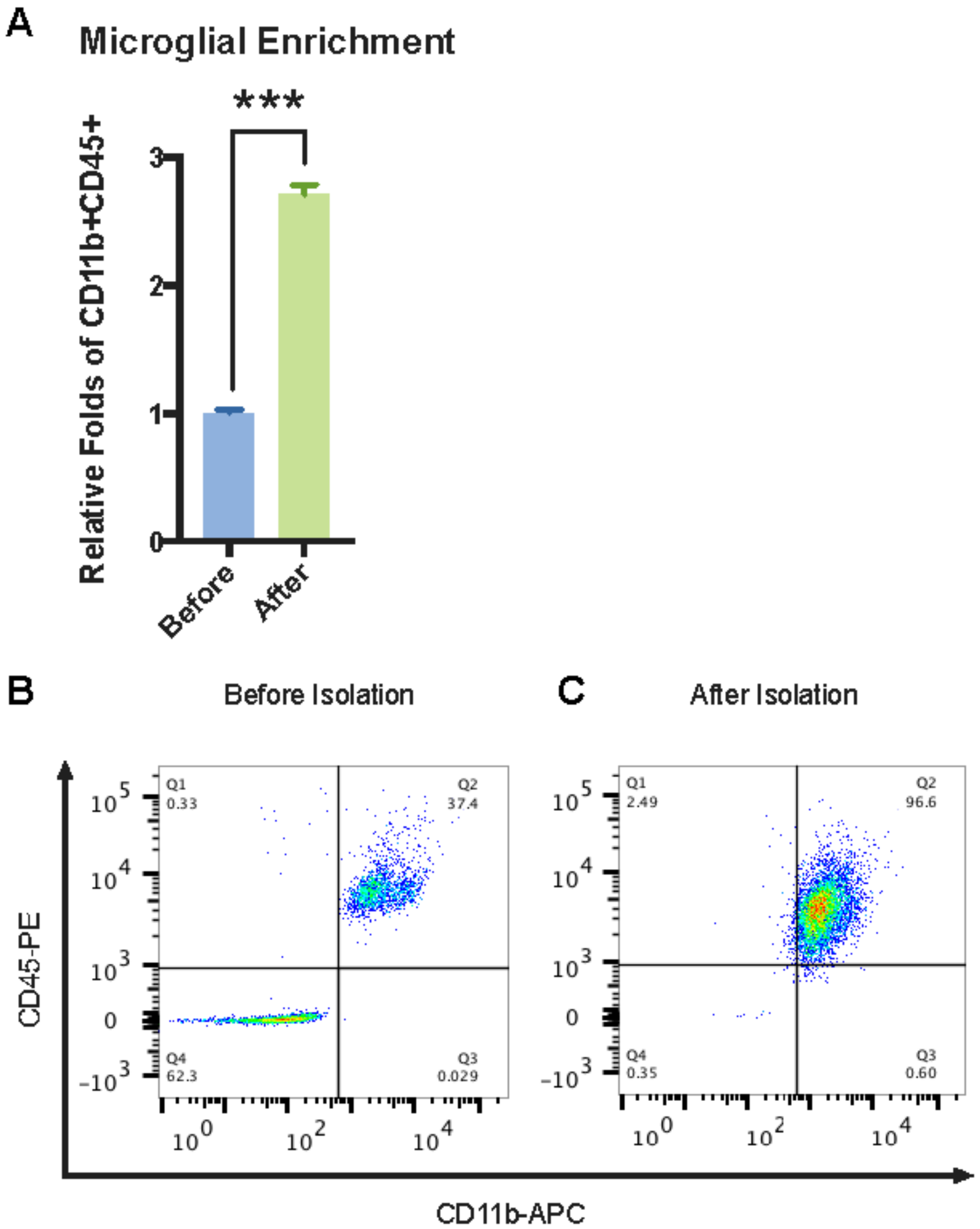
A
Microglial Enrichment
***
Relative Folds of CD11b+CD45+
3
2
1
0
Before
After
B
Before Isolation
C
After Isolation
CD45-PE
CD11b-APC
Q1 0.33
Q2 37.4
Q4 62.3
Q3 0.029
Q1 2.49
Q2 96.6
Q4 0.35
Q3 0.60
